# Supplementary material for: Modeling bulk mechanical effects in a planar cellular monolayer
Source: Phys Rev E. Author manuscript; Available in PMC 2026 Aug 20. (PMC7619381; doi:10.1103/5l56-wc8f)
Supplement: Supplementary file 1 [file EMS217245-supplement-Appendix.pdf]

material stiffness arises from nonzero second derivatives of U with respect to A, L, and H: the full set of nine second derivatives contribute resistance to out-of-plane shear deformation in (H3). Stiffness also arises from prestress [associated with first derivatives of U in (H3), for example]. For in-plane deformations, (H5) shows how prestress associated with cortical tension is lost at a rigidity transition; this is illustrated by fragmentation of the networks shown in Fig. 9(d), leading to isolated patches of stiffness as rigidity is lost. We characterized this by counting zero eigenvalues of a vertex Laplacian (F5) and the connected components of the associated graph. An important caveat of the analysis in Appendix H is that a monolayer of disordered cells in a rigid state can be expected to undergo nonaffine deformations under an external load, and nonaffine effects become exaggerated in the floppy state, as illustrated in Fig. 12. Computations of deformations under simple loads (Fig. 7) illustrate the strongly nonlinear anisotropic response arising from the combined linear and quadratic functions in the chosen mechanical energy. For the chosen energies, cells can become columnar under modest external compression (Fig. 7) or under modest parameter variation (Fig. 6).

We grew disordered monolayers by allowing a sequence of random cell divisions, followed by relaxation to the nearest equilibrium. This generates in-plane stresses that, in the rigid regime, cannot be resolved via neighbor exchanges. Our simulations show evidence of elevated in-plane compression towards the center of the monolayer, which promotes local cell elongation (Fig. 8). It will be helpful to assess these findings against 3D simulations that do not assume apical-basal symmetry, and which allow more fine-grained variations of height of the apical vertices.

In summary, the present model shows how 3D bulk mechanical effects can be incorporated in a 2D modeling framework, even when accommodating an out-of-plane force balance. The model has the flexibility to capture independent variations in cell aspect ratio and monolayer stiffness, to describe in-plane and bulk stresses, and it demonstrates how long-range cell height variations can arise in a growing tissue.

ACKNOWLEDGMENTS

This work was supported by The Leverhulme Trust (RPG-2021-394) and a Wellcome Trust Career Development Award (225408/Z/22/Z).

DATA AVAILABILITY

The data that support the findings of this article are openly available [43].

APPENDIX A: INCIDENCE AND ADJACENCY MATRICES

Assigning orientations to all quantities, signed incidence matrices A, B, and C with components $C_{hi} = \{\mathsf{C}\}_{hi}$, $B_{ij} = \{\mathsf{B}\}_{ij}$, and $A_{jk} = \{\mathsf{A}\}_{jk}$ define topological arrangements. Specifically, an edge orientation is defined by a tangent with magnitude equal to the edge length; a face orientation is defined by a normal with magnitude equal to the face area; $C_{hi} = 1$ (-1) if the normal to face i points into (out of) cell h; $B_{ij} = 1$ (-1) if edge j bounds face i and is congruent (or anticongruent) to its orientation in a right-handed sense; $A_{jk} = 1$ (-1) if edge j points into (out of) vertex k. The components of A, B, and C are zero otherwise.

A includes a subblock A^{++} connecting N_e^+ apical edges to N_v^+ apical vertices; B has subblocks B^{++} connecting N_f^+ apical faces to N_e^+ apical edges and B^{l+} connecting N_f^l lateral faces to N_e^+ apical edges; and C has blocks C^+ and C^l connecting N_c cells to N_f^+ apical and N_f^l lateral faces, respectively. Thus

$$\mathsf{A} = \begin{pmatrix} \mathsf{A}^{++} & 0 \\ \mathsf{A}^{l+} & \mathsf{A}^{l-} \\ 0 & \mathsf{A}^{--} \end{pmatrix}, \tag{A1a}$$

$$\mathsf{B} = \begin{pmatrix} \mathsf{B}^{++} & 0 & 0 \\ \mathsf{B}^{l+} & \mathsf{B}^{ll} & \mathsf{B}^{l-} \\ 0 & 0 & \mathsf{B}^{--} \end{pmatrix}, \tag{A1b}$$

$$\mathsf{C} = \begin{pmatrix} \mathsf{C}^+ & \mathsf{C}^l & \mathsf{C}^- \end{pmatrix}. \tag{A1c}$$

The requirements that $\mathsf{CB} = 0$ and $\mathsf{BA} = 0$, arising because the 3D network is bounded by closed faces, imply

$$\begin{pmatrix} \mathsf{C}^+\mathsf{B}^{++} + \mathsf{C}^l\mathsf{B}^{l+} & \mathsf{C}^l\mathsf{B}^l & \mathsf{C}^-\mathsf{B}^{--} + \mathsf{C}^l\mathsf{B}^{l-} \end{pmatrix} = 0, \tag{A2a}$$

$$\begin{pmatrix} \mathsf{B}^{++}\mathsf{A}^{++} & 0 \\ \mathsf{B}^{l+}\mathsf{A}^{++} + \mathsf{B}^{ll}\mathsf{A}^{l+} & \mathsf{B}^{l-}\mathsf{A}^{--} + \mathsf{B}^{ll}\mathsf{A}^{l-} \\ 0 & \mathsf{B}^{--}\mathsf{A}^{--} \end{pmatrix} = 0. \tag{A2b}$$

The apical matrices $\mathsf{C}^+\mathsf{B}^{++}$ and A^{++} are the 3D analogs of the face-edge and edge-vertex incidence matrices used in prior 2D studies [35,39,44]; they satisfy the requirement that $(\mathsf{C}^+\mathsf{B}^{++})\mathsf{A}^{++} = 0$. A 2D topological cell Laplacian is then

$$\mathcal{L} \equiv -\mathsf{C}^+\mathsf{B}^{++}\mathsf{E}\mathsf{B}^{++\top}\mathsf{C}^{+\top}. \tag{A3}$$

This is regularized, as in [44], by taking E to be a diagonal $N_e \times N_e$ matrix for which $E_{jj} = 1$ unless j labels a peripheral apical edge of a peripheral cell in an isolated monolayer, in which case $E_{jj} = 0$. This regularization ensures that $\mathcal{L}\mathsf{1}_c = 0$, where $\mathsf{1}_c = (1, 1, \dots)$ is the chain identifying all cells in a monolayer.

The adjacency matrices connecting vertices, edges, faces, and cells are

$$D^{\rm cv}_{hk} \equiv \frac{1}{6}\sum_{i,j} |C_{hi}||B_{ij}||A_{jk}|, \quad D^{\rm ev}_{jk} \equiv |A_{jk}|, \tag{A4a}$$

$$D^{\rm ce}_{hj} \equiv \frac{1}{2}\sum_{i} |C_{hi}||B_{ij}|, \quad D^{\rm fe}_{ij} \equiv |B_{ij}|, \tag{A4b}$$

$$D^{\rm fv}_{ik} \equiv \frac{1}{2}\sum_{j} |B_{ij}||A_{jk}|, \quad D^{\rm cf}_{hi} \equiv |C_{hi}|. \tag{A4c}$$

The factor $\frac{1}{6}$ in $\mathsf{D}^{\rm cv}$ accounts for the fact that each vertex k of cell h neighbors three edges of the cell, and each edge bounds two faces of the cell. For the prismatic cells illustrated Fig. 1, distinguishing apical $(+)$, lateral (l), and basal $(-)$ objects, the adjacency matrices inherit the structure of the incidence matrices via

$$\mathsf{D}^{\rm fv} = \begin{pmatrix} \mathsf{D}^{\rm fv++} & 0 \\ \mathsf{D}^{{\rm fv},l+} & \mathsf{D}^{{\rm fv},l-} \\ 0 & \mathsf{D}^{\rm fv--} \end{pmatrix}, \tag{A5a}$$

$$\mathsf{D}^{\rm ce} = (\mathsf{D}^{\rm ce+} \quad \mathsf{D}^{{\rm ce},l} \quad \mathsf{D}^{\rm ce-}), \tag{A5b}$$

$$\mathsf{D}^{\rm cv} = (\mathsf{D}^{\rm cv+} \quad \mathsf{D}^{\rm cv-}). \tag{A5c}$$

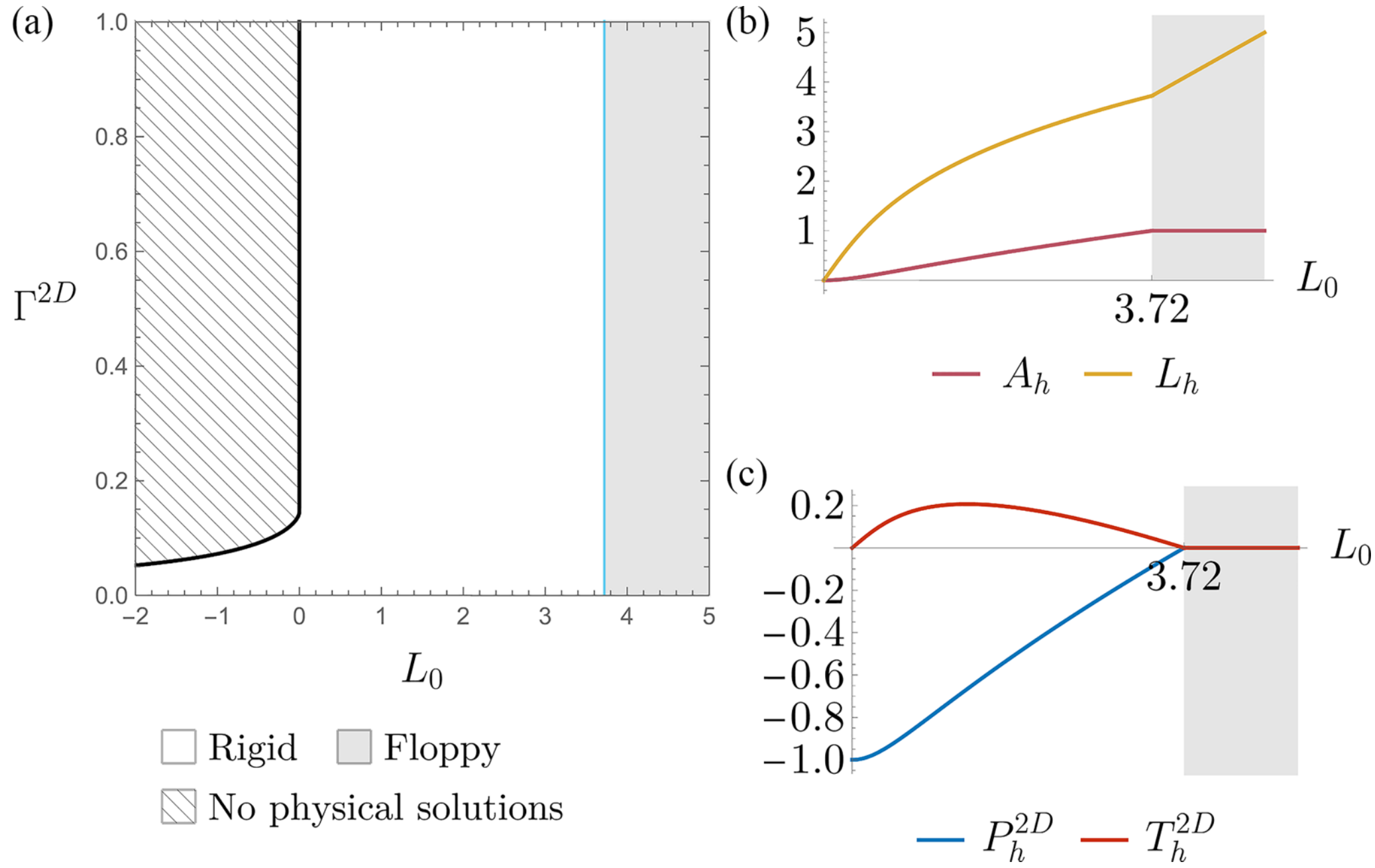

FIG. 10. (a) The (L_0, Γ^{2D})-parameter map of the classical 2D vertex model [1]. Cells are rigid in the white region, becoming floppy for $L_0 > s_6 \approx 3.72$. No solutions exist in the hatched region. (b) Area A_h and perimeter L_h, and (c) pressure P_h^{2D} and tension T_h^{2D}, are plotted as functions of L_0 for $\Gamma = 0.24$; when $L_0 = 1$, A_h, L_h take the baseline values in Table I.

APPENDIX B: GEOMETRIC IDENTITIES

Given vertex locations $\mathbf{r}_k^{\pm}$, we construct apical cell edge tangents and centroids using

$$\mathbf{t}_j^+ = \sum_j A_{jk}^{++}\mathbf{r}_k^+, \quad \mathbf{c}_j^+ = \frac{1}{2}\sum_j |A_{jk}^{++}|\mathbf{r}_k^+, \tag{B1}$$

respectively. The apical perimeter and apical area of cell h are then, respectively, for $h = 1, \dots, N_c$,

$$L_h^+ = \sum_{i,j} |C_{hi}^l||B_{ij}^{l+}||\mathbf{t}_j^+|, \tag{B2a}$$

$$A_h^+ = \frac{1}{2}\sum_{i,j} |C_{hi}^+|B_{ij}^{++}\mathbf{c}_j^+ \cdot (\mathbf{t}_j^+ \times \hat{\mathbf{n}}_{hi}^+), \tag{B2b}$$

where $\hat{\mathbf{n}}_{hi}^+ \equiv \hat{\mathbf{z}}$; $\hat{\mathbf{n}}_{hi}$ is the unit outward normal to face i of cell h. The area (B2b) mirrors results in [35,39]. To capture the manner in which the apical perimeter changes as a result of displacing an apical vertex, we use the relations

$$\frac{\partial t_i^+}{\partial \mathbf{r}_k^+} = \sum_j |B_{ij}^{l+}|A_{jk}^{++}\hat{\mathbf{t}}_j^+, \tag{B3a}$$

$$\frac{\partial L_h^+}{\partial \mathbf{r}_k^+} = \sum_{i,j} |C_{hi}^l||B_{ij}^{l+}|A_{jk}^{++}\hat{\mathbf{t}}_j^+ = \sum_i |C_{hi}^l|\frac{\partial t_i^+}{\partial \mathbf{r}_k^+}, \tag{B3b}$$

where the hat denotes a unit vector.

APPENDIX C: 2D MODEL

The classical 2D model is recovered from the present formulation by imposing $H = 1$ in (8) (disregarding the vertical force balance), setting $\Gamma_a = 0$, and defining $\Gamma^{2D} = 2\Gamma_L$ and $L_0^{2D} = L_0 - \Gamma_A/(4\Gamma_L)$. This gives

$$U_h = \tfrac{1}{2}(A_h - 1)^2 + \tfrac{1}{2}\Gamma^{2D}\big(L_h - L_0^{2D}\big)^2 + \text{const.} \tag{C1}$$

The associated pressure and tensions are

$$P_h^{2D} \equiv \frac{\partial U_h}{\partial A_h} = A_h - 1, \quad T_h^{2D} \equiv \frac{\partial U_h}{\partial L_h} = \Gamma^{2D}\big(L_h - L_0^{2D}\big). \tag{C2}$$

Rigid hexagons satisfy (16a) and (16b) (with $a_0 = 1$); floppy solutions satisfy $A_h = 1$, $L_h = L_0^{2D}$, as illustrated in Figs. 10(b) and 10(c) (replicating [1], for example), which can be compared to Fig. 2. The parameter space map in Fig. 10(a) can be compared to Fig. 6(c). The rigid-floppy transition lies at $L_0^{2D} = s_6 \approx 3.72$. Rigid solutions collapse to zero area along $L_0^{2D} = 0$ for $\Gamma^{2D} > \Gamma^* \equiv \sqrt{3}/12$. Rigid solution branches terminate via a saddle-node bifurcation at $L_0^{2D} = -[8\sqrt{2}/(3\Gamma)](\Gamma^* - \Gamma^{2D})^{\frac{3}{2}}$, with $0 < \Gamma^{2D} \leqslant \Gamma^*$.

APPENDIX D: HEIGHT VARIATION ACROSS A CONSTRAINED MONOLAYER

In the main presentation of the model, we impose the requirement that $\mathbf{r}^{\pm}$ and H evolve independently. Alternatively, this constraint can be enforced by redefining the total energy to accommodate small variations in height between adjacent cells. We write $\mathsf{H} = (H_1, \dots, H_{N_c})^\top$ and redefine U as

$$U = \sum_h \mathcal{U}(A_h, L_h, H_h) + \frac{1}{2}\Lambda \mathsf{H}^\top \mathcal{L}\mathsf{H} \tag{D1}$$

[see Eq. (8)]. The Laplacian term [see Eq. (A3)] mimics an external constraint that penalizes height differences between neighboring cells, when the constant Λ is positive. Then, noting that $\mathcal{L} = \mathcal{L}^\top$, the vertical force balance on individual

cells $\partial U/\partial H_h = 0$ becomes

$$0 = \frac{\partial \mathcal{U}}{\partial H}(A_h, L_h, H_h) + \Lambda\{\mathcal{L}\mathsf{H}\}_h. \quad \text{(D2)}$$

Assuming $\Lambda \gg 1$ and writing $\mathsf{H} = \mathsf{H}^{(0)} + \mathsf{H}^{(1)}/\Lambda + \cdots$ at leading order it follows that $\mathcal{L}\mathsf{H}^{(0)} = \mathsf{0}$. This has a solution for which $\mathsf{H}^{(0)}$ is uniform across the monolayer, with $H_h^{(0)} = H^*$, say, for $h = 1, 2, \ldots, N_c$. At the following order,

$$\{\mathcal{L}\mathsf{H}^{(1)}\}_h = -\frac{\partial \mathcal{U}}{\partial H}(A_h, L_h, H^*). \quad \text{(D3)}$$

As $\mathcal{L}\mathsf{1} = 0$, so that $\mathsf{1}^\top\mathcal{L} = \mathsf{0}^\top$, it follows that a solvability condition for (D3) is that $\sum_h(\partial\mathcal{U}/\partial H)(A_h, L_h, H^*) = 0$, which is equivalent to the global force balance (12). We can then evaluate small corrections to cell height by inverting (D3).

APPENDIX E: ASYMPTOTIC LIMITS

Here we identify solutions of (14a), (14b), and (16) describing configurations of rigid hexagonal cells and floppy cells in specific asymptotic limits.

1. Constrained total surface area

For $\Gamma_a \gg 1$, which constrains the total cell surface area to take the target value of unity, we reformulate the problem for rigid hexagons as (16a) and (16b), with

$$0 = 2A + LH - 1, \quad \text{(E1a)}$$

$$P^{2\mathrm{D}} = H(AH - 1) + 2T, \quad \text{(E1b)}$$

$$0 = A(AH - 1) + \left(T + \tfrac{1}{2}\Gamma_A\right)L, \quad \text{(E1c)}$$

$$T^{2\mathrm{D}} = TH + \tfrac{1}{2}\Gamma_A H + 2\Gamma_L(L - L_0). \quad \text{(E1d)}$$

Here the tension T is introduced as a Lagrange multiplier, and (E1d) derives from (14c) using T from (9b) (second expression). The total area constraint (E1a) can be used to eliminate H; the vertical force balance (E1c) specifies T; and $P^{2\mathrm{D}}$ and $T^{2\mathrm{D}}$ are then combined in (16a) and (16b) to find l. Within this limit, columnar cells satisfy

$$L \approx \frac{1}{H}, \quad A \approx \frac{\sqrt{3}a_0^3}{24H^2}, \quad T \approx -\frac{1}{2}\Gamma_A + \frac{\sqrt{3}a_0^3}{24H}, \quad \text{(E2a)}$$

$$P^{2\mathrm{D}} \approx -H, \quad T^{2\mathrm{D}} \approx \frac{\sqrt{3}a_0^3}{12}, \quad L_0 \approx -\frac{\sqrt{3}a_0^3}{48\Gamma_L}, \quad \text{(E2b)}$$

with $H \gg 1$. Here the tension T balances adhesive forces. Floppy states under large Γ_a are obtained by solving (E1) with $P^{2\mathrm{D}} = 0$, $T^{2\mathrm{D}} = 0$ for A, H, L, and T, which accommodates the solution for elongated cells

$$A = 0, \quad H = 1/L_0, \quad L = L_0, \quad T = -\tfrac{1}{2}\Gamma_A, \quad \text{(E3)}$$

shown in Figs. 3(a) and 3(c). Imposing (19) on floppy states yields the large-Γ_a limits shown in Figs. 6(b), 6(d), 6(e), and 14(a).

2. Constrained apical perimeter

For $\Gamma_L \gg 1$, the apical perimeter is constrained to satisfy $L = L_0$, and we express the problem as

$$A = \left(\sqrt{3}a_0^3/24\right)L_0^2, \quad \text{(E4a)}$$

$$P^{2\mathrm{D}} = H(AH - 1) + 2\Gamma_a(2A + L_0H - 1), \quad \text{(E4b)}$$

$$0 = A(AH - 1) + \left[\Gamma_a(2A + L_0H - 1) + \tfrac{1}{2}\Gamma_A\right]L_0, \quad \text{(E4c)}$$

$$T^{2\mathrm{D}} = \Gamma_a(2A + L_0H - 1)H + \tfrac{1}{2}\Gamma_A H + T^c. \quad \text{(E4d)}$$

In this case, the hexagonal constraint (E4a) determines A, the vertical force balance (E4c) specifies H, and the Lagrange multiplier T^c takes whatever value is needed to satisfy the force balance (16a) and (16b). This leads to asymptotes shown in Fig. 5. Floppy solutions satisfying (17) in this limit are recovered by solving (E4b)–(E4d) with $P^{2\mathrm{D}} = 0$, $T^{2\mathrm{D}} = 0$ for A, H, and T^c (and $L = L_0$). Imposing (19) in addition leads to limits plotted in Figs. 6(c), 6(d), 6(f), and 14(c). Setting $H = 0$ in (E4a) and (E4c) leads to

$$\Gamma_A = \frac{\sqrt{3}a_0^3}{12}L_0 + \Gamma_a\left(2 - \frac{\sqrt{3}a_0^3}{6}L_0^2\right), \quad \text{(E5)}$$

while (E4a) and (E4c) in the limit $H \to \infty$ requires

$$\Gamma_a = -L_0^3a_0^6/192. \quad \text{(E6)}$$

3. Strong adhesion

The vertical force balance (16c) can be written as $0 = A(AH - 1) + [\Gamma_a(2A + LH - 1) + \frac{1}{2}\Gamma_A]L$, and it is then combined with (14b) to become $0 = HA(AH - 1) + L[T^{2\mathrm{D}} - 2\Gamma_L(L - L_0)]$. Then, in the limit $\Gamma_A \to -\infty$,

$$H \approx \frac{2\Gamma_a^2 - \Gamma_L}{2\Gamma_a(\Gamma_a - \Gamma_L L_0)}\Gamma_A, \quad L \approx \frac{\Gamma_a - \Gamma_L L_0}{2\Gamma_a^2 - \Gamma_L}, \quad \text{(E7a)}$$

$$A \approx \frac{2\Gamma_a\Gamma_L(2\Gamma_a L_0 - 1)(\Gamma_a - \Gamma_L L_0)}{\Gamma_A\left(2\Gamma_a^2 - \Gamma_L\right)^2}. \quad \text{(E7b)}$$

This represents the dominant balance $0 \approx H(AH - 1) + 2\Gamma_a LH$ in (14a), $0 \approx \Gamma_a LH^2 + \frac{1}{2}\Gamma_A H$ in (14b), and $AH(AH - 1) - 2\Gamma_L(L - L_0) = 0$ in the vertical direction. The tension balance is similar to the rigid limit (E9), but the deviation from the target volume is balanced in the pressure condition by tension associated with the area of lateral faces, and in the vertical force balance by peripheral apical tension. At a rigidity transition, cells become hexagonal, and the geometric constraint (19) cannot be met unless

$$L_0 \approx \Gamma_a/\Gamma_L \quad (-\Gamma_A \to \infty). \quad \text{(E8)}$$

This limit is shown in Figs. 6(a), 6(e), and 6(f).

Additionally, for $\Gamma_A \to -\infty$, analysis of (14a), (14b), and (16) reveals the limit

$$A \approx \frac{96\Gamma_a^2}{\sqrt{3}a_0^3\Gamma_A^2}, \quad L \approx \frac{48\Gamma_a}{\sqrt{3}a_0^3(-\Gamma_A)}, \quad H \approx \frac{\sqrt{3}a_0^3\Gamma_A^2}{96\Gamma_a^2}, \quad \text{(E9)}$$

satisfying the dominant balance $AH - 1 \approx 0$ in (14a), $\Gamma_a LH^2 + \frac{1}{2}\Gamma_A H \approx 0$ in (14b) and (16c), with the area and perimeter satisfying the isoperimetric constraint (16b); each

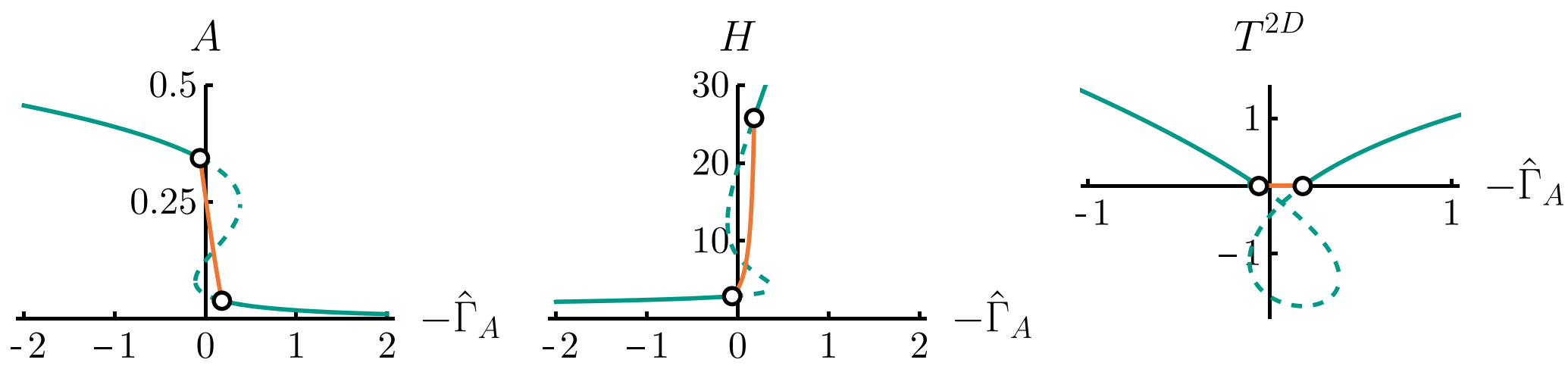

FIG. 11. Example illustrating a sharp squamous-to-columnar transition when cell volume is constrained. Green curves show solutions of (E10) for $a_0 = 7$, $\hat{\Gamma}_L \equiv \Gamma_L/\Gamma_a = 0.1$ for varying $-\hat{\Gamma}_A \equiv -\Gamma_A/\Gamma_a$. Orange lines show floppy states satisfying (E10a) and (E10c)–(E10e) with $P^{2D} = T^{2D} = 0$ for which $A \leqslant \sqrt{3}a_0^3 L^2/24$. Symbols indicate the two bifurcation points where the floppy and rigid solution branches intersect and rigid states lose stability (solid curves become dashed).

cell is close to its target volume, and an inward tension associated with total surface area, which is dominated by lateral faces, balances the outward tension due to adhesion.

4. Constrained volume

Here we consider

$$H = 1/A, \tag{E10a}$$

$$A = \sqrt{3}a_0^3 L^2/24, \tag{E10b}$$

$$0 = AP + [\Gamma_a(2A + LH - 1) + \tfrac{1}{2}\Gamma_A]L, \tag{E10c}$$

$$P^{2D} = HP + 2\Gamma_a(2A + LH - 1), \tag{E10d}$$

$$T^{2D} = \Gamma_a(2A + LH - 1)H + \tfrac{1}{2}\Gamma_A H + 2\Gamma_L(L - L_0), \tag{E10e}$$

$$0 = 2AP^{2D} + LT^{2D} \tag{E10f}$$

in the limit $\Gamma_a \sim \Gamma_A \sim \Gamma_L \ll 1$. We include P as a Lagrange multiplier enforcing the volume constraint $AH = 1$. [Floppy states satisfy (E10a), and (E10c)–(E10e) with $P^{2D} = T^{2D} = 0$ and (18).] Setting $\Gamma_L = 0$, scaling P, P^{2D}, and T^{2D} on Γ_A, and setting $\hat{\Gamma}_A = \Gamma_A/\Gamma_a$, Eq. (E10) reduces to

$$(9l^3 - 2)[\sqrt{3}(9l^3 + 4) - 3a_0 l] = 3\hat{\Gamma}_A l a_0, \tag{E11}$$

where $l = \frac{1}{6}a_0^2 L$. The left-hand side of (E11) is a product of two cubics: the first has a single zero at $\ell = (2/9)^{1/3}$ in $\ell > 0$; the second cubic (proportional to $2A + LH - 1$) has two positive zeros for $a_0 > 2^{2/3}3^{7/6} \approx 5.71$. These zeros also first appear at $\ell = (2/9)^{1/3}$, implying that ℓ becomes a multivalued function of γ for $a_0 > 2^{2/3}3^{7/6}$. Floppy states for $\Gamma_L = 0$ and $a_0 > 2^{2/3}3^{7/6}$ exist along $\hat{\Gamma}_A = 0$, connecting roots of (E11) for which $2A + LH = 1$. Thus, as predicted by [31] using a slightly different model, the squamous-to-columnar transition with $\Gamma_L = 0$ is abrupt and takes place at $\hat{\Gamma}_A = 0$.

For nonzero Γ_L, however, the branch of floppy solutions bends away from $\Gamma_A = 0$ and intersects the branches of rigid solutions at new locations. Figure 11 illustrates one example. The transition is continuous, but a branch of floppy states connects the locally stable rigid squamous and columnar states.

APPENDIX F: DYNAMICS

We consider dynamic evolution of a monolayer under the forces identified in (11). Following [35], we define $\mathsf{s}(\mathbf{r}) = (A_1, \dots, A_{N_c}, L_1, \dots, L_{N_c})^\top$ and collect energy derivatives as $U_\mathsf{s} \equiv (P_1^{2D}, \dots, P_{N_c}^{2D}, T_1^{2D}, \dots, T_{N_c}^{2D})^\top$. Imposing a drag on vertices, time evolution of the system follows

$$\begin{pmatrix} 1 & 0 \\ 0 & \mu \end{pmatrix} \frac{\mathrm{d}}{\mathrm{d}t} \begin{pmatrix} \mathbf{r} \\ H \end{pmatrix} = -\begin{pmatrix} U_\mathsf{s}\mathsf{s}_\mathbf{r}, \\ U_H \end{pmatrix}. \tag{F1}$$

Here the parameter μ models frictional resistance to height changes, relative to drag forces that resist in-plane vertex motion, and subscripts on U denote derivatives. The energy can be expanded as

$$U \approx \bar{U} + (\bar{U}_\mathsf{s}\bar{\mathsf{s}}_\mathbf{r}, \bar{U}_H)\begin{pmatrix} \cdot\hat{\mathbf{r}} \\ \hat{H} \end{pmatrix} + \frac{1}{2}(\hat{\mathbf{r}}\cdot, \hat{H})\begin{pmatrix} \bar{\mathsf{s}}_\mathbf{r}^\top \bar{U}_{\mathsf{ss}}\bar{\mathsf{s}}_\mathbf{r} + \bar{U}_\mathsf{s}{}^\top \bar{\mathsf{s}}_{\mathbf{rr}} & \bar{\mathsf{s}}_\mathbf{r}^\top \bar{U}_{\mathsf{s}H} \\ \bar{U}_{\mathsf{s}H}^\top \bar{\mathsf{s}}_\mathbf{r} & \bar{U}_{HH} \end{pmatrix}\begin{pmatrix} \cdot\hat{\mathbf{r}} \\ \hat{H} \end{pmatrix} + \cdots. \tag{F2}$$

A bar denotes evaluation at an equilibrium point (so that $\bar{U}_\mathsf{s} = \mathsf{0}$, $\bar{U}_H = 0$) and hats denote small perturbations. Provided $\bar{U}_{HH} \neq 0$, rapid relaxation of the depth [linearizing (F1) about the equilibrium and taking $\mu \to 0$] leads to the eigenvalue problem

$$\lambda\hat{\mathbf{r}} = \left[\bar{\mathsf{s}}_\mathbf{r}^\top\left(\bar{U}_{\mathsf{ss}} - \bar{U}_{\mathsf{s}H}\bar{U}_{HH}^{-1}\bar{U}_{\mathsf{s}H}^\top\right)\bar{\mathsf{s}}_\mathbf{r} + \bar{U}_\mathsf{s}{}^\top \bar{\mathsf{s}}_{\mathbf{rr}}\right]\cdot\hat{\mathbf{r}}, \tag{F3}$$

where the solutions λ are the decay rates of small perturbation. The matrix operator in (F3) is the Hessian $\mathcal{H}$.

Figure 12 shows the values of the nonzero eigenvalues of $\mathcal{H}$ for an isolated hexagonal cell, for varying L_0, Γ_a, and Γ_A, as discussed in Sec. III. In all cases, all symmetry-breaking modes simultaneously become unstable when $T^{2D} = 0$. The cell height vanishes at $\Gamma_A \approx 1.42$ when Γ_A is varied. All other unstable points correspond to a loss of in-plane rigidity. Eigenvalues of the symmetry-breaking modes diverge to $\pm\infty$ when the equilibrium cell area and perimeter go to zero.

For some parameter values, multiple equilibrium solutions for rigid hexagonal cells can coexist; the Hessian in (F3) was used to confirm the stability of solutions plotted in Fig. 6.

The component of the Hessian $\bar{U}_{\bar{\mathsf{s}}}^\top \mathsf{s}_{\mathbf{rr}}$ in (F3) contains contributions from prestress through the vector $\bar{U}_\mathsf{s}$ of equilibrium pressures $\bar{\mathsf{P}}^{2D}$ and tensions $\bar{\mathsf{T}}^{2D}$. $\bar{\mathsf{s}}_{\mathbf{rr}}$ contains derivatives of (B3). Focusing on components relating to $\bar{\mathsf{T}}^{2D}$, we define an indicator function satisfying

$$\mathcal{I}_j^\varepsilon = \begin{cases} 1 & \left|\sum_h D_{hj}^{\mathrm{ce}}\bar{T}_h^{2D}\right| > \varepsilon, \\ 0 & \text{otherwise,} \end{cases} \quad (h = 1, \dots, N_c), \tag{F4}$$

which identifies edges carrying tension above a threshold. Evaluation of the derivative $\partial^2 L_h^+/\partial\mathbf{r}_k^+\partial\mathbf{r}_{k'}^+$ (see Ref. [35]) and

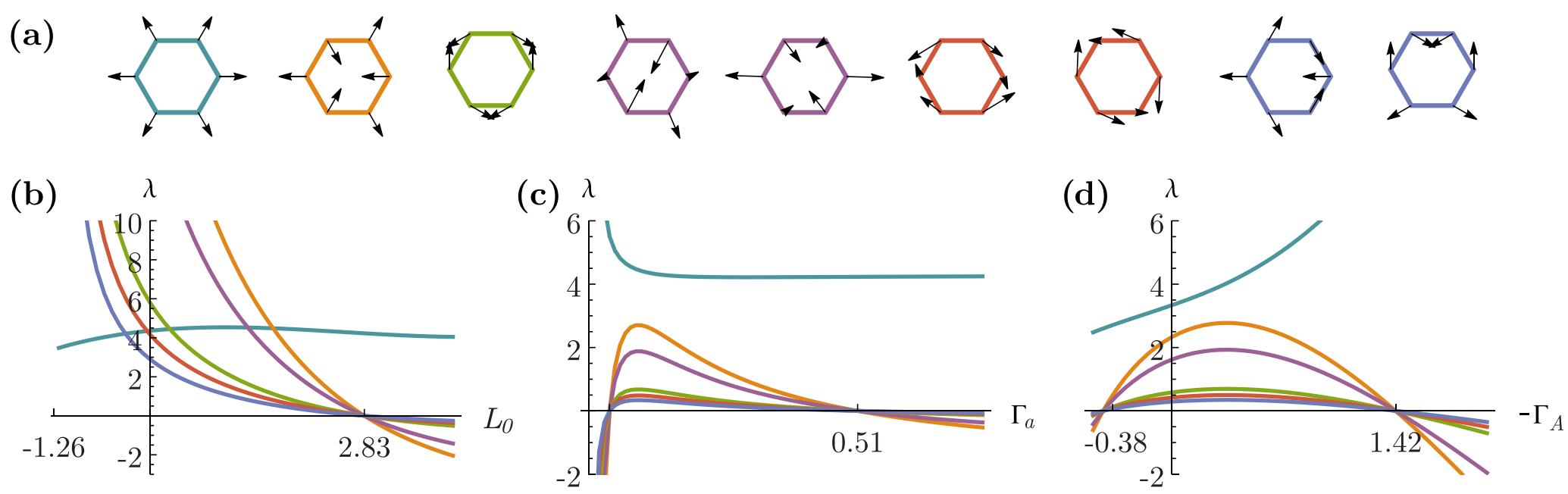

FIG. 12. Eigenmodes (a) and eigenvalues of the Hessian $\mathcal{H}$ as (b) L_0, (c) Γ_a, and (d) $-\Gamma_A$ are varied, for a hexagon at baseline parameter values (Table I). Eigenvalues correspond to the mode(s) of the same color in (b)–(d); modes of the same color in (a) share an eigenvalue. The dilation mode is shown in cyan. Translation and rotation modes (with zero eigenvalue) are not shown.

multiplication by $\mathcal{I}_h^\varepsilon$ leads to consideration of the operator

$$\{\mathcal{L}^\varepsilon\}_{kk'} \equiv \sum_j \mathcal{I}_j^\varepsilon A_{jk}^{++} A_{jk'}^{++}. \tag{F5}$$

This modification of the full apical vertex Laplacian $\mathsf{A}^{++\top}\mathsf{A}^{++}$ retains contributions from edges of cells carrying nonvanishing tension.

APPENDIX G: STRESS

The stress for the symmetric cells considered here can be derived directly by using the energy (8) and writing the force on vertex k as

$$\begin{aligned}\mathbf{f}_{hk} &= \frac{\partial U}{\partial A_h}\frac{\partial A_h}{\partial \mathbf{R}_k} + \frac{\partial U}{\partial L_h^+}\frac{\partial L_h^+}{\partial \mathbf{R}_k} + \frac{\partial U}{\partial L_h^-}\frac{\partial L_h^-}{\partial \mathbf{R}_k} + \frac{1}{Z_h}\frac{\partial U}{\partial H}\frac{\partial H}{\partial \mathbf{R}_k}\\ &= \left(H\frac{\partial U}{\partial V_h} + 2\frac{\partial U}{\partial a_h}\right)\frac{\partial A_h}{\partial \mathbf{R}_k}\\ &\quad + \left(H\frac{\partial U}{\partial a_h} + T_h^c + \frac{1}{2}\Gamma_A H\right)\frac{\partial L_h}{\partial \mathbf{R}_k} + \frac{1}{Z_h}\frac{\partial U}{\partial H}\frac{\partial H}{\partial \mathbf{R}_k},\end{aligned} \tag{G1}$$

where $\partial U/\partial H = A_h \partial U/\partial V_h + L_h \partial U/\partial a_h + \frac{1}{2}\Gamma_A L_h$. Here Z_h is the number of apical vertices, across which the vertical force is distrubuted. We then exploit 2D results [38], plus the observation that $\mathbf{R}_k \otimes (\partial H/\partial \mathbf{R}_k) = H\hat{\mathbf{z}} \otimes \hat{\mathbf{z}}$ when we enforce $\mathbf{R}_k = \mathbf{r}_k$ for vertices in the plane $z = 0$, and $\mathbf{R}_k = \mathbf{r}_k + H\hat{\mathbf{z}}$ for cells in the plane $z = H$, to derive (20).

APPENDIX H: STIFFNESS

Consider the energy $\mathcal{U}(A, L, H)$ for a single cell (8). In this appendix, h suffixes are dropped, and subscripts on $\mathcal{U}$ denote derivatives. 2D and 3D stresses (20) can be written

$$V\boldsymbol{\sigma} = A\boldsymbol{\sigma}^{2D} + H\mathcal{U}_H \hat{\mathbf{z}} \otimes \hat{\mathbf{z}}, \quad A\boldsymbol{\sigma}^{2D} = A\mathcal{U}_A \mathsf{I}_\perp + L\mathcal{U}_L \mathsf{Q}. \tag{H1}$$

Recall that $\mathsf{I}_\perp : \mathsf{Q} = 1$, $2AP^{\text{eff},2D} = 2A\mathcal{U}_A + L\mathcal{U}_L$ and $3VP^{\text{eff}} = 2A\mathcal{U}_A + L\mathcal{U}_L + H\mathcal{U}_H$. For a symmetric hexagonal cell, $\mathsf{Q} = \frac{1}{2}\mathsf{I}_\perp$. We then impose an affine deformation $\mathsf{E} = \mathsf{E}_\perp + e\hat{\mathbf{z}} \otimes \hat{\mathbf{z}}$. For example, isotropic expansion has $\mathsf{E}_\perp = e\mathsf{I}$ so that $\mathrm{Tr}(\mathsf{E}) = 3e$; in-plane shear deformation has $e = 0$ with $\mathsf{I}_\perp : \mathsf{E}_\perp = 0$; out-of-plane shear deformation has $\mathsf{E}_\perp = -\frac{1}{2}e\mathsf{I}_\perp$. We now seek the stress generated by an affine displacement; this approach complements that of [20], who evaluated the elastic modulus of a cell modeled in 3D as a truncated octahedron.

Under an arbitrary small deformation, perturbations in A, L, H, and Q are $\Delta A = A\mathsf{I}_\perp : \mathsf{E}$, $\Delta L = L\mathsf{Q} : \mathsf{E}$, $\Delta H = He$, and $\Delta\mathsf{Q} = \mathsf{B} : \mathsf{E}$, respectively, where the fourth-order tensor B (defined in [45]) satisfies $\mathsf{B} : \mathsf{I}_\perp = \mathsf{Q}$ and $\mathrm{Tr}(\mathsf{B} : \mathsf{E}) = \mathsf{Q} : \mathsf{E}$. Perturbing all quantities in (H1) and linearizing leads to

$$V\Delta\boldsymbol{\sigma} = \boldsymbol{\alpha}(\mathsf{I}_\perp : \mathsf{E}_\perp) + \boldsymbol{\beta}e + \boldsymbol{\gamma}\mathsf{Q} : \mathsf{E}_\perp + \delta\mathsf{B} : \mathsf{E}_\perp, \tag{H2a}$$

$$\begin{aligned}\boldsymbol{\alpha} &= A^2\mathcal{U}_{AA}\mathsf{I}_\perp + (LA\mathcal{U}_{LA} - L\mathcal{U}_L)\mathsf{Q}\\ &\quad + (AH\mathcal{U}_{AH} - H\mathcal{U}_H)\hat{\mathbf{z}} \otimes \hat{\mathbf{z}},\end{aligned} \tag{H2b}$$

$$\begin{aligned}\boldsymbol{\beta} &= (AH\mathcal{U}_{AH} - A\mathcal{U}_A)\mathsf{I}_\perp + (LH\mathcal{U}_{LH} - L\mathcal{U}_L)\mathsf{Q}\\ &\quad + H^2\mathcal{U}_{HH}\hat{\mathbf{z}} \otimes \hat{\mathbf{z}},\end{aligned} \tag{H2c}$$

$$\boldsymbol{\gamma} = AL\mathcal{U}_{AL}\mathsf{I}_\perp + (L^2\mathcal{U}_{LL} + L\mathcal{U}_L)\mathsf{Q} + HL\mathcal{U}_{HL}\hat{\mathbf{z}} \otimes \hat{\mathbf{z}}, \tag{H2d}$$

$$\delta = L\mathcal{U}_L. \tag{H2e}$$

These coefficients contain second derivatives of the energy, contributing to material stiffness, plus first derivatives, contributing to geometric stiffness. The first derivatives can be identified as prestresses in the baseline state in (H1). Equation (H2) demonstrates how the cross-coupling in $\mathcal{U}$ leading to nonzero mixed second derivatives can influence the cell stiffness.

The stress-strain relationship under isotropic expansion is then

$$\begin{aligned}V\Delta\boldsymbol{\sigma} &= [2\boldsymbol{\alpha} + \boldsymbol{\beta} + \boldsymbol{\gamma} + \delta\mathsf{Q}]e\\ &= \{(2A^2\mathcal{U}_{AA} + AL\mathcal{U}_{AL} + AH\mathcal{U}_{AH} - A\mathcal{U}_A)\mathsf{I}_\perp\\ &\quad + (2LA\mathcal{U}_{AL} + L^2\mathcal{U}_{LL} + LH\mathcal{U}_{HL} - L\mathcal{U}_L)\mathsf{Q}\\ &\quad + (2AH\mathcal{U}_{AH} + HL\mathcal{U}_{HL} + H^2\mathcal{U}_{HH} - 2H\mathcal{U}_H)\hat{\mathbf{z}} \otimes \hat{\mathbf{z}}\}e.\end{aligned} \tag{H3a}$$

For a hexagonal cell, this simplifies to

$$V\Delta\boldsymbol{\sigma} = [2\boldsymbol{\alpha} + \boldsymbol{\beta} + \boldsymbol{\gamma} + \delta\mathsf{Q}]e \tag{H4a}$$

$$\begin{aligned}&= \{[2A^2\mathcal{U}_{AA} + 2AL\mathcal{U}_{AL} + AH\mathcal{U}_{AH} - A\mathcal{U}_A\\ &\quad + \tfrac{1}{2}(L^2\mathcal{U}_{LL} + LH\mathcal{U}_{HL} - L\mathcal{U}_L)]\mathsf{I}_\perp\\ &\quad + (2AH\mathcal{U}_{AH} + HL\mathcal{U}_{HL} + H^2\mathcal{U}_{HH} - 2H\mathcal{U}_H)\hat{\mathbf{z}} \otimes \hat{\mathbf{z}}\}e.\end{aligned} \tag{H4b}$$

The trace of (H4) recovers a bulk modulus, illustrated more directly in Fig. 8(c). The anisotropic response to isotropic

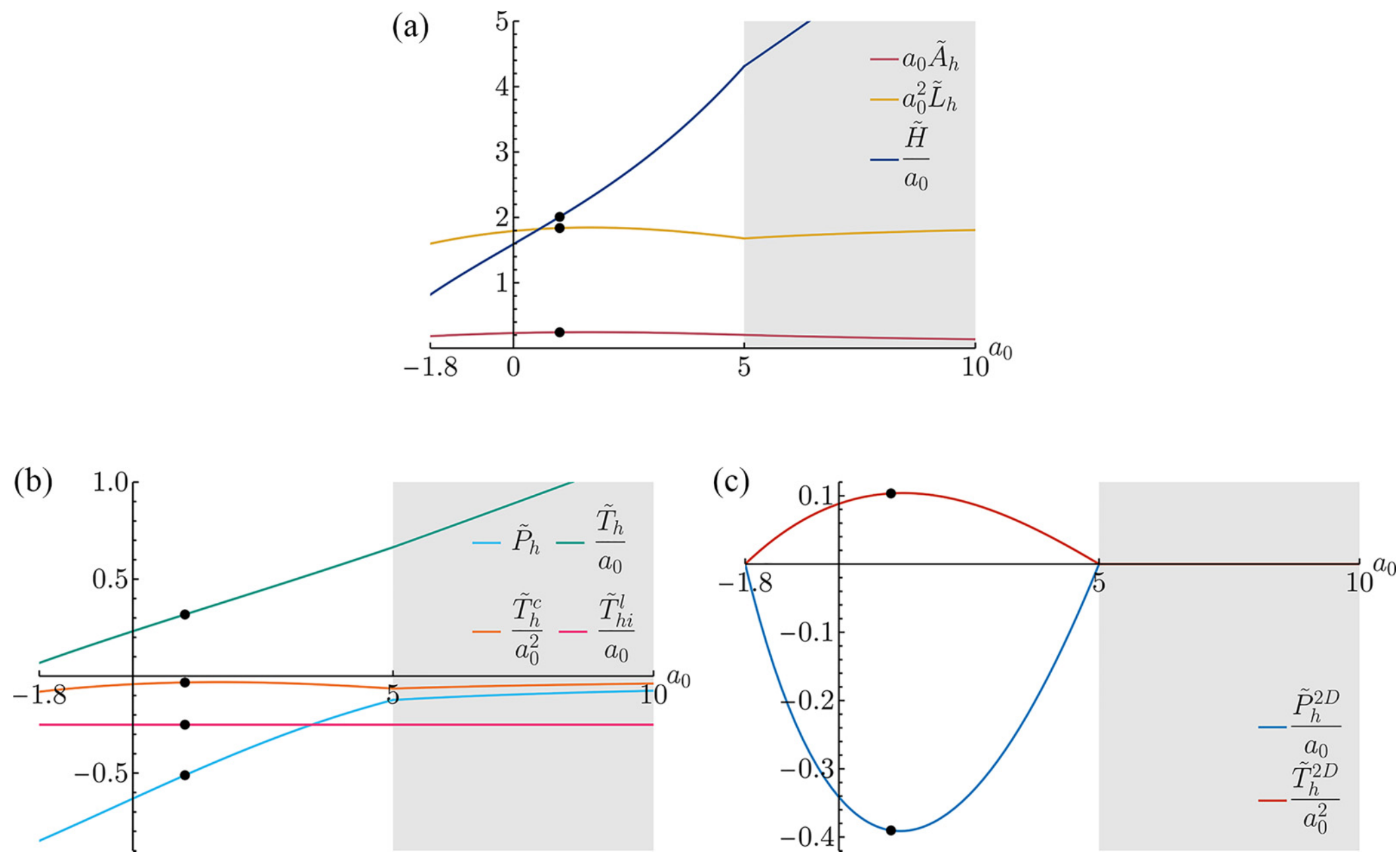

FIG. 13. Impact of varying a_0, presented following the format of Fig. 2, except that geometric variables are plotted without implementing the rescaling in (13).

forcing, evident in Fig. 8, is consistent with different in-plane and out-of-plane stress responses in (H4).

In-plane shear deformation leads to

$$V\Delta\boldsymbol{\sigma} = [AL\mathcal{U}_{AL}\mathsf{I}_\perp + (L^2\mathcal{U}_{LL} + L\mathcal{U}_L)\mathsf{Q} + HL\mathcal{U}_{HL}\hat{\mathbf{z}}\otimes\hat{\mathbf{z}}]\mathsf{Q} : \mathsf{E}_\perp + L\mathcal{U}_L\mathsf{B} : \mathsf{E}_\perp. \quad \text{(H5)}$$

Two terms proportional to $L\mathcal{U}_L$ rely on cortical tension to generate stiffness. In contrast, for the classical 2D model with $\mathcal{U} = \frac{1}{2}(A-1)^2 + \frac{1}{2}\Gamma(L-L_0)^2$ and $T \equiv \mathcal{U}_L$, (H5) simplifies to

$$V\Delta\boldsymbol{\sigma} = \Gamma L^2\mathsf{Q}(\mathsf{Q} : \mathsf{E}_\perp) + LT[\mathsf{Q}(\mathsf{Q} : \mathsf{E}_\perp) + \mathsf{B} : \mathsf{E}_\perp]. \quad \text{(H6)}$$

The first term provides material stiffness (via $\mathcal{U}_{LL} = \Gamma$), the second geometric stiffness (via $U_L = T$). In (H5), additional material stiffness arises from U_{AL} and U_{HL}.

Finally, out-of-plane shear deformation leads to

$$\begin{aligned} V\Delta\boldsymbol{\sigma} &= \left[-\boldsymbol{\alpha} + \boldsymbol{\beta} - \tfrac{1}{2}\boldsymbol{\gamma} - \tfrac{1}{2}\delta\mathsf{Q}\right]e \\ &= \Big\{\big(-A^2\mathcal{U}_{AA} - \tfrac{1}{2}AL\mathcal{U}_{AL} + AH\mathcal{U}_{AH} - A\mathcal{U}_A\big)\mathsf{I}_\perp \\ &\quad + \big(-LA\mathcal{U}_{AL} - \tfrac{1}{2}L^2\mathcal{U}_{LL} + LH\mathcal{U}_{HL} - L\mathcal{U}_L\big)\mathsf{Q} \\ &\quad + \big(-AH\mathcal{U}_{AH} - \tfrac{1}{2}HL\mathcal{U}_{HL} + H^2\mathcal{U}_{HH} + H\mathcal{U}_H\big)\hat{\mathbf{z}} \\ &\quad \otimes\hat{\mathbf{z}}\Big\}e. \end{aligned} \quad \text{(H7)}$$

For a hexagonal cell, this simplifies to

$$\begin{aligned} V\Delta\boldsymbol{\sigma} &= \left[-\boldsymbol{\alpha} + \boldsymbol{\beta} - \tfrac{1}{2}\boldsymbol{\gamma} - \tfrac{1}{2}\delta\mathsf{Q}\right]e \\ &= \Big\{\big(-A^2\mathcal{U}_{AA} - AL\mathcal{U}_{AL} + AH\mathcal{U}_{AH} - A\mathcal{U}_A \\ &\quad - \tfrac{1}{4}L^2\mathcal{U}_{LL} + \tfrac{1}{2}LH\mathcal{U}_{HL} - \tfrac{1}{2}L\mathcal{U}_L\big)\mathsf{I}_\perp \\ &\quad + \big(-AH\mathcal{U}_{AH} - \tfrac{1}{2}HL\mathcal{U}_{HL} + H^2\mathcal{U}_{HH} + H\mathcal{U}_H\big)\hat{\mathbf{z}} \\ &\quad \otimes\hat{\mathbf{z}}\Big\}e, \end{aligned} \quad \text{(H8)}$$

showing how all second derivatives of U contribute to the material stiffness.

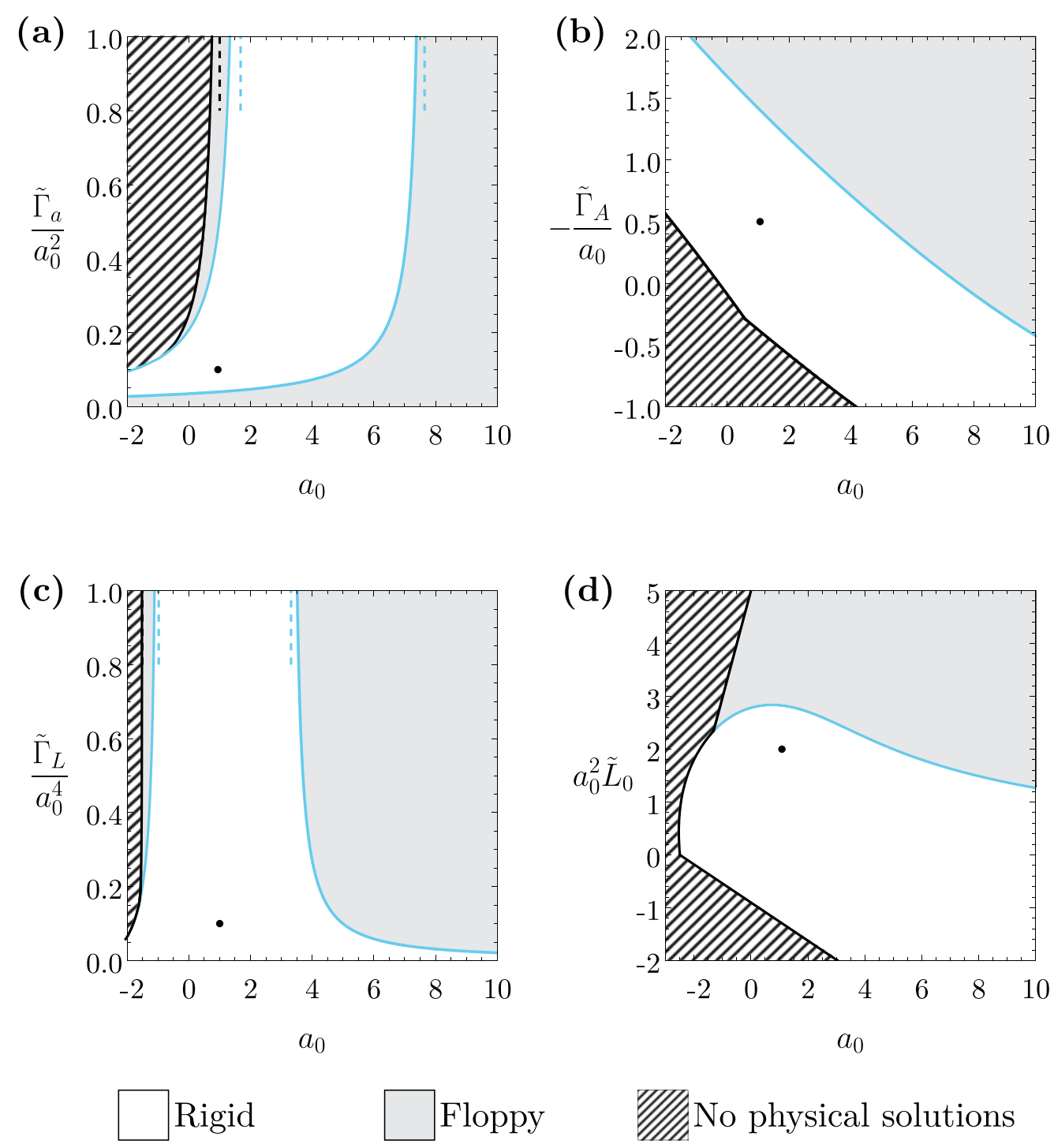

FIG. 14. Cross-sections of a_0 with $(L_0, \Gamma_a, -\Gamma_A, \Gamma_L)$-parameter space, intersecting the baseline case (black circle). Parameters are plotted without implementing the rescaling in (13). In each plot, only two parameters are varied. Hatching shows regions where there are no physical solutions. Gray regions show the floppy regime. Blue solid lines show where $T^{2D} = 0$, corresponding to a rigidity transition. Blue dashed lines show asymptotes (E1) and (19) with $P^{2D} = T^{2D} = 0$ in (a), and (E4) with $P^{2D} = T^{2D} = 0$ in (c). The black dashed line in (a) shows (E3).

APPENDIX I: TARGET SURFACE AREA

The effects of varying the parameter a_0 are shown in Figs. 13 and 14. Both figures show variables and parameters without implementing the scaling by a_0 in (13). Axes and legend labels are given in terms of the scaled values used elsewhere but with the tilde explicitly shown here.

[1] R. Farhadifar, J.-C. Röper, B. Aigouy, S. Eaton, and F. Jülicher, The influence of cell mechanics, cell-cell interactions, and proliferation on epithelial packing, Curr. Biol. **17**, 2095 (2007).

[2] P. Gómez-Gálvez, P. Vicente-Munuera, S. Anbari, J. Buceta, and L. M. Escudero, The complex three-dimensional organization of epithelial tissues, Development **148**, dev195669 (2021).

[3] T. Sego, T. Comlekoglu, S. M. Peirce, D. W. Desimone, and J. A. Glazier, General, open-source vertex modeling in biological applications using tissue forge, Sci. Rep. **13**, 17886 (2023).

[4] S. Runser, R. Vetter, and D. Iber, SimuCell3D: 3D simulation of tissue mechanics with cell polarization, Nat. Comput. Sci. **4**, 299 (2024).

[5] H. Honda, M. Tanemura, and T. Nagai, A three-dimensional vertex dynamics cell model of space-filling polyhedra simulating cell behavior in a cell aggregate, J. Theor. Biol. **226**, 439 (2004).

[6] S. Okuda, Y. Inoue, M. Eiraku, Y. Sasai, and T. Adachi, Reversible network reconnection model for simulating large deformation in dynamic tissue morphogenesis, Biomech. Model. Mechanobiol. **12**, 627 (2013).

[7] O. M. Drozdowski and U. S. Schwarz, Morphological instability at topological defects in a three-dimensional vertex model for spherical epithelia, Phys. Rev. Res. **6**, L022045 (2024).

[8] E. Hannezo, J. Prost, and J.-F. Joanny, Theory of epithelial sheet morphology in three dimensions, Proc. Natl. Acad. Sci. USA **111**, 27 (2014).

[9] P. Sahu, J. Schwarz, and M. L. Manning, Geometric signatures of tissue surface tension in a three-dimensional model of confluent tissue, New J. Phys. **23**, 093043 (2021).

[10] C. Villeneuve, A. Hashmi, I. Ylivinkka, E. Lawson-Keister, Y. A. Miroshnikova, C. Pérez-González, S.-M. Myllymäki, F. Bertillot, B. Yadav, T. Zhang *et al.*, Mechanical forces across compartments coordinate cell shape and fate transitions to generate tissue architecture, Nat. Cell Biol. **26**, 207 (2024).

[11] M. Merkel and M. L. Manning, A geometrically controlled rigidity transition in a model for confluent 3D tissues, New J. Phys. **20**, 022002 (2018).

[12] F. Ioannou, M. A. Dawi, R. J. Tetley, Y. Mao, and J. J. Muñoz, Development of a new 3D hybrid model for epithelia morphogenesis, Front. Bioeng. Biotechnol. **8**, 405 (2020).

[13] A. K. Khan, G. Muñoz-Castro, and J. J. Muñoz, Single and two-cells shape analysis from energy functionals for three-dimensional vertex models, Int. J. Num. Meth. Biomed. Eng. **39**, e3766 (2023).

[14] C. H. Durney and J. J. Feng, A three-dimensional vertex model for drosophila salivary gland invagination, Phys. Biol. **18**, 046005 (2021).

[15] X. Du, M. Osterfield, and S. Y. Shvartsman, Computational analysis of three-dimensional epithelial morphogenesis using vertex models, Phys. Biol. **11**, 066007 (2014).

[16] N. Noll, S. J. Streichan, and B. I. Shraiman, Variational method for image-based inference of internal stress in epithelial tissues, Phys. Rev. X **10**, 011072 (2020).

[17] G. Ogita, T. Kondo, K. Ikawa, T. Uemura, S. Ishihara, and K. Sugimura, Image-based parameter inference for epithelial mechanics, PLoS Comput. Biol. **18**, e1010209 (2022).

[18] D. Bi, J. Lopez, J. Schwarz, and M. Manning, A density-independent rigidity transition in biological tissues, Nat. Phys. **11**, 1074 (2015).

[19] T. Zhang and J. M. Schwarz, Topologically-protected interior for three-dimensional confluent cellular collectives, Phys. Rev. Res. **4**, 043148 (2022).

[20] K. Kim, T. Zhang, and J. Schwarz, Mean-field elastic moduli of a three-dimensional, cell-based vertex model, New J. Phys. **26**, 043009 (2024).

[21] Y. Mao and B. Baum, Tug of war—the influence of opposing physical forces on epithelial cell morphology, Dev. Biol. **401**, 92 (2015).

[22] T. Kondo and S. Hayashi, Mechanisms of cell height changes that mediate epithelial invagination, Dev. Growth Differ. **57**, 313 (2015).

[23] J. M. Gomez, Y. Wang, and V. Riechmann, Tao controls epithelial morphogenesis by promoting fasciclin 2 endocytosis, J. Cell Biol. **199**, 1131 (2012).

[24] R. P. Ray, P. S. Ganguly, S. Alt, J. R. Davis, A. Hoppe, N. Tapon, G. Salbreux, and B. J. Thompson, Apical and basal matrix remodeling control epithelial morphogenesis, Dev. Cell **46**, 23 (2018).

[25] P. Khandelwal, S. N. Abraham, and G. Apodaca, Cell biology and physiology of the uroepithelium, Am. J. Phys. - Renal Phys. **297**, F1477 (2009).

[26] S. Harmansa and T. Lecuit, Mechanical regulation of cuboidal-to-squamous epithelial transition in the *Drosophila* developing wing, bioRxiv 2024.09.21.614249 (2024), doi: https://doi.org/10.1101/2024.09.21.614249.

[27] S. J. Chisolm, E. Guo, V. Subramaniam, K. D. Schulze, and T. E. Angelini, Elastic modulus versus cell packing density in mdck epithelial monolayers, J. Biomech. **184** 112648 (2025).

[28] V. Barone, A. Tagua, J. Á. Román, A. Hamdoun, J. Garrido-García, D. C. Lyons, and L. M. Escudero, Local and global changes in cell density induce reorganisation of 3d packing in a proliferating epithelium, Development **151**, dev202362 (2024).

[29] P. Gómez-Gálvez, P. Vicente-Munuera, A. Tagua, C. Forja, A. M. Castro, M. Letrán, A. Valencia-Expósito, C. Grima, M. Bermúdez-Gallardo, Ó. Serrano-Pérez-Higueras *et al.*, Scutoids are a geometrical solution to three-dimensional packing of epithelia, Nat. Commun. **9**, 1 (2018).

[30] J. Rozman, M. Krajnc, and P. Ziherl, Basolateral mechanics prevents rigidity transition in epithelial monolayers, Phys. Rev. Lett. **133**, 168401 (2024).

[31] T. Sarkar and M. Krajnc, Reentrant rigidity transition in planar epithelia with volume-and area elasticity, arXiv:2505.12558.

[32] M. De Marzio, A. Das, J. J. Fredberg, and D. Bi, Epithelial layer fluidization by curvature-induced unjamming, Phys. Rev. Lett. **134**, 138402 (2025).
